# A Cross-Sectional Study of Endogenous Antioxidants and Patterns of Dental Visits of Periodontitis Patients

**DOI:** 10.3390/ijerph16020180

**Published:** 2019-01-09

**Authors:** Chang-Yu Lee, Cheuk-Sing Choy, Yu-Cheng Lai, Chao-Chien Chang, Nai-Chia Teng, Wan-Ting Huang, Che-Tong Lin, Yung-Kai Huang

**Affiliations:** 1 Division of Periodontics, Department of Dentistry, Taipei Medical University Hospital, Taipei Medical University, Taipei 110, Taiwan; m8504005@tmu.edu.tw; 2 Department of Community Medicine, En Chu Kong Hospital, New Taipei City 237, Taiwan; prof.choy@gmail.com; 3 Department of Nursing, Yuanpei University of Medical Technology, Hsinchu 300, Taiwan; 4 Department of Dentistry, En Chu Kong Hospital, New Taipei City 237, Taiwan; peter90637@hotmail.com; 5 Department of Pharmacology, School of Medicine, College of Medicine, Taipei Medical University, Taipei 110, Taiwan; change@seed.net.tw; 6 Division of Cardiology, Department of Internal Medicine, Cathay General Hospital, Taipei 106, Taiwan; 7 School of Dentistry, College of Oral Medicine, Taipei Medical University; Taipei 110, Taiwan; dianaten@tmu.edu.tw (N.-C.T.); m204102005@tmu.edu.tw (W.-T.H.); chetong@tmu.edu.tw (C.-T.L.); 8 School of Oral Hygiene, College of Oral Medicine, Taipei Medical University, Taipei 110, Taiwan; 9 Department of Oral Hygiene, College of Dental Medicine, Kaohsiung Medical University, Kaohsiung 80708, Taiwan

**Keywords:** salivary biomarker, dental visit, oxidative stress response, periodontitis, prevention

## Abstract

Periodontitis is an inflammatory disease, wherein endogenous antioxidants help to balance the inflammatory status. Oral health behaviors are related to the periodontal disease status. The aim of this study was to explore the associations between oral health behaviors and endogenous antioxidants in periodontitis patients. In total, 225 subjects diagnosed with periodontitis were enrolled in the study. Information obtained from the initial interview included socioeconomic and demographic characteristics, lifestyle factors, and oral health-related behaviors. The clinical periodontal parameters evaluated included bleeding on probing (BOP), the plaque index (PI), and probing depth (PD). Stimulated saliva was collected before periodontal therapy to determine five endogenous antioxidants (copper-zinc superoxide dismutase (Cu/Zn SOD), manganese SOD (MnSOD), thioredoxin 1 (Trx1), peroxiredoxin 2 (Prx2), and catalase (CAT)). When these five factors were adjusted for in patients whose last previous dental visit was >1 year, the patients’ PI, BOP, and PD showed significant decreases because of an elevation in the Cu/Zn SOD level. Associations of endogenous antioxidants with levels of clinical periodontal parameters were much higher in subjects whose last previous dental visit was >1 year, compared to subjects whose last previous dental visit was <1 year. This study provides a better understanding of dental visit patterns and the salivary endogenous antioxidants that may underlie the symptomatic development of preclinical periodontitis.

## 1. Introduction

Periodontal disease is one of the most important global oral health burdens [1]. In the US, 47% of adults are affected by periodontal diseases [2]. It was estimated that approximately 90% of adults had mild to severe periodontitis in Taiwan [3]. The prevalence of periodontal disease has significantly increased in Taiwan over the past 17 years [4]. Compared to other countries, such as India (72%), Italy (35~40%), and the US (47%), the high prevalence of periodontitis in Taiwan is noteworthy [5,6,7].

Periodontitis is a chronic inflammatory disease that is caused by a pathogenic microbiotic infection, which triggers the innate immune system [8]. Periodontal pathogenic infections cause inflammation and oxidative stress in the body and lead to the destruction of periodontal tissues; therefore, eliminating excess oxidative stress can retard the disease activity of periodontitis [9]. Periodontal disease is preventable by controlling risk factors, such as low frequencies of dental visits and professional cleaning, diabetes, smoking, and poor oral hygiene behaviors [10,11,12]. All of the risk factors for periodontitis are related to inflammation. One study also showed an association between oxidative stress and systemic inflammation in people with severe periodontitis [9].

The antioxidant system includes enzymatic and non-enzymatic antioxidants which are effective in neutralizing oxidative damage [13]. Antioxidants balance the oxidative stress which originates from pathogen-induced inflammation. Enzymatic antioxidants (endogenous antioxidants) are found in all cells of eukaryotic organisms, protect organisms from oxidative damage, and play roles in balancing the periodontal inflammatory status [14]. The three main endogenous antioxidants of biological systems are superoxide dismutase (SOD), catalase (CAT), and glutathione peroxidase (GPx) [15]. When reactive oxygen species (ROS) are generated, the thioredoxin (Trx) system is stimulated and transduces redox signals to alter the activities of antioxidant enzymes, such as SOD, CAT, and GPx to eliminate free radicals. Dismutation of superoxide radicals (O_2_^•-^) which are released from ROS is catalyzed by SOD, which turns them into H_2_O_2_. H_2_O_2_ is scavenged by CAT, peroxiredoxin 2 (Prx2), and GPx/glutathione, by turning it into H_2_O and O_2_. Hydroxyl radicals (OH•) formed by splitting H_2_O_2_ can cause DNA and protein damage and lipid peroxidation [16]. Specific inflammatory biomarkers are valuable in periodontal disease diagnoses and evaluating the disease progression [17]. The total oxidant status and SOD levels can be evaluated, and these reflect the severity of the disease and the effectiveness of periodontal treatment [18].

Compared to other biological fluids, the main advantages of saliva are the ease and noninvasiveness of its collection. Saliva is secreted from salivary glands and is a complex biological fluid that reflects the physical health status and disease pathological status [19]. Under normal conditions, saliva plays active protective roles in oral health. Previous studies showed that salivary SOD and the total antioxidant capacity of healthy subjects were higher than those of periodontitis patients [20]. SOD was correlated with many inflammatory diseases and could reflect the onset of disease [21]. In an animal model, the level of dental plaque decreased when rats were treated with an antioxidant-containing diet [22]. The endogenous antioxidant capacity may be related to periodontitis.

Periodontitis is a multifactorial disease, simply resulting from inadequate tooth brushing or irregular dental visits, but it can become a severe life-threatening systemic disease. Inadequate tooth brushing leads to plaque accumulation which covers all surfaces of the teeth and inside the gingival sulcus. Regular dental visits also play an important role in preventing periodontitis. A previous meta-analysis study showed that fair and poor oral hygiene significantly increased the periodontal disease risk by two- and five-fold, respectively [11]. In Taiwan, a 52% increase in the number of patients who received periodontal disease medical treatment was recorded over the last decade [3]. Most periodontitis subjects had poor oral hygiene and had ignored warning signs because their symptoms were not obvious. Poor knowledge of preclinical disease symptoms and the fear of dental visits resulted in patients not undergoing regular dental checkups. This phenomenon showed that subjects used periodontal disease treatment rather than preventive periodontal services. In order to prevent periodontal disease, salivary endogenous antioxidants reflect the physical health status and can serve as an auxiliary tool to elevate disease awareness in subjects who are in the preclinical phase of periodontal disease. Thus, it is important to focus on oral hygiene behaviors and the physical status (endogenous antioxidants) of periodontitis patients. The hypothesis of this study was that oral health behaviors may influence salivary endogenous antioxidants in periodontitis patients. The aim of this study was to explore the associations between oral hygiene behaviors and endogenous antioxidants in periodontitis patients.

## 2. Materials and Methods

**Figure 1:**
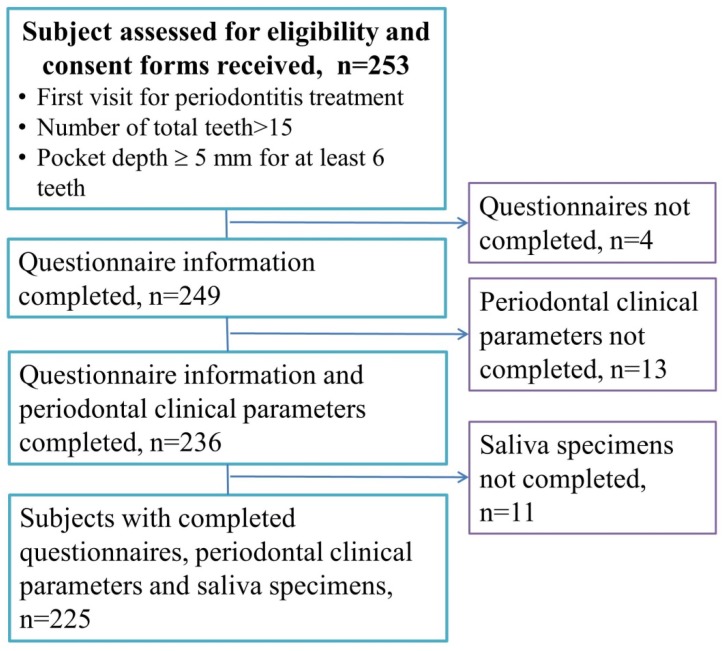
Flowchart of the participant enrollment.

This was a cross-sectional study. Participants were enrolled from the Division of Periodontal, Department of Dentistry, Taipei Medical University Hospital between July 2013 and April 2016. Figure 1 is a flowchart of the participant enrollment. We invited 253 periodontitis patients to participate in the study, and 249 patients completed the informed consent form. After the patients had completed the questionnaire information, periodontal clinical parameters, and saliva specimens, 225 patients were recruited. No subjects had made regular dental visits at 6 months, and all the subjects were eligible for the comprehensive periodontal treatment project (CTPT). The CTPT is a National Health Insurance (NHI) program to reduce periodontal disease in Taiwan [23]. The criteria of the CTPT are as follows: the number of functional teeth must be >15, and the probing depth should be ≥5 mm for at least six teeth. Patients who had received periodontal therapy, or were pregnant, or had been diagnosed with cancer were excluded from the study. This study was approved by the Research Ethics Committee of the Taipei Medical University Joint Institutional Review Board (date of approval: 15 June 2013; approval no.: TMU-JIRB no. 201303021), and it complied with the World Medical Association’s *Declaration of Helsinki*.

Experienced assistants conducted a standardized personal interview based on a structured questionnaire after the subjects had signed the consent form. Information obtained from the interview included socioeconomic and demographic characteristics, lifestyle factors, and oral health-related behaviors. Oral health behaviors included the pattern of dental visits, tooth cleaning frequency, and the use of any auxiliary tooth-cleaning tools (dental floss or an interdental brush). Information on the individual and family medical histories, such as inflammatory diseases and cardiovascular diseases, was requested using the structured questionnaire and medical records.

To reduce the inter- and intra-variations in clinical parametric assessments, all clinical examinations were carried out by the same periodontist. This periodontist followed the examiner alignment and assessment procedures in periodontal research published by Hefti and Preshaw [24], to perform all the clinical parametric assessments in this study. Data on the plaque index (PI), bleeding on probing (BOP), and probing depth (PD) were collected as the clinical parameters. The PI measurement was based on both soft debris and mineralized deposits on four (buccal, lingual, mesial, and distal) surfaces of a tooth, and the presence or absence of plaque was recorded for all sites [25]. The BOP and PD were measured using a periodontal probe at six (distobuccal, buccal, mesiobuccal, distolingual, lingual, and mesiolingual) sites on each tooth. In this study, BOP was presented as a periodontitis inflammatory index. The index of BOP was presented as a percentage, and the percentage of BOP was the number of bleeding sites divided by the number of all the sites probed. The mean depth of all sites of PD (PD mean) and the percentage of PD sites that were >4 mm (percentage of sites with a PD of ≥4 mm) were presented as a measure of the severity of the periodontal condition. PI, BOP, and PD were the clinical periodontal parameters.

Sample size estimation was based on the previous study in Reference [26] and the G*Power software (correlation, point biserial model) [27]. We assumed that the ρ of the SOD and PI was 0.35, alpha of 0.05, a power of 0.90, and two tails. Based on the aforementioned assumptions, the desired sample size was 78. Three crucial clinical indicators in periodontitis are BOP, PI, and PD. Studies have suggested that there were no significant differences in these three indicators between subjects whose last previous dental visit was 6 months or 1 year [28,29,30]. In this study, the length of time since the last dental visit was determined from the questionnaire responses to these questions: Question 1. When was the last time you had a dental checkup? (1) Within 1 year; (2) 1~2 years; (3) 2~5 years; (4) 5~10 years; (5) More than 10 years. Question 2. What is the reason that you did not have a dental checkup within the past 1 year? (1) A dental checkup was performed within the past 1 year; (2) Not able to afford the fee; (3) Had an unpleasant experience before; (4) Simply no time for a dental checkup; (5) Feeling great with no problems with my oral condition. When a patient answered (1) Within 1 year for question 1 and (1) Dental checkup was performed within the past 1 year for question 2, they were categorized as a subject whose last dental visit was <1 year previous (75 subjects). The rest of the subjects were placed in the group whose last dental visit was >1 year previous (150 subjects).

Stimulated saliva specimens were collected by asking a subject to chew paraffin wax for 5 min using a Saliva-Check kit (GC Corporation, Tokyo, Japan), before the subject received the first periodontal treatment. A protease inhibitor cocktail (Roche Applied Science, Mannheim, Germany) was added to the saliva samples at a ratio of 1 mL saliva: 10 µL of protease inhibitor cocktail. After adding the protease inhibitor, the saliva specimen was centrifuged (3000 rpm) for 3 min at room temperature, and the supernatant was collected to determine the oxidative stress biomarkers. Levels of five biomarkers of oxidative stress, including Cu/Zn SOD, manganese SOD (MnSOD), CAT, thioredoxin 1 (Trx1), and peroxiredoxin 2 (Prx2), were determined using a MILLIPLEX^®^ MAP Human Oxidative Stress Magnetic Bead Panel kit (Merck Millipore, Darmstadt, Germany). Utilizing internally color-coded microspheres with two fluorescent dyes and precise concentrations of the dyes, distinctly colored beads were created and coated with a specific capture antibody. For this procedure, each sample (35 μL) was diluted to an identical quantity of protein (10 µg) with assay buffer, added to a 96-well plate, mixed with 35 μL of assay buffer and 25 μL of antibody-immobilized beads, and then incubated for 2 h at room temperature, followed by incubation with detection antibodies (50 μL) and streptavidin phycoerythrin (50 μL). The mean fluorescence intensity was determined, and the concentrations of the five oxidative stress biomarkers were calculated by comparing the fluorescence units to a calibration curve (0.55, 1.65, 4.94, 14.81, 44.44, 133.33, and 400 µg/mL). The *R*-squared value for the standard curve was >0.995. The coefficient of variance (CV) for the intra-assay ranged between 3.49~8.10%, and the CV for the inter-assay ranged between 1.22% and 12.30%.

Data analyses were carried out using SAS software (vers. 9.4; SAS Corp., Cary, NC, USA). The student’s *t*-test and an analysis of variance (ANOVA) were used to compare the periodontal clinical parameters and salivary endogenous antioxidants among demographic characteristics and oral health behaviors. Simple and multiple general linear regression model analyses were performed to examine the relationships of oral health behaviors and endogenous antioxidants with the periodontal clinical parameters. The variance inflation factor (VIF) was used to check the multicollinearity of the salivary antioxidants, conventional factors, and oral health behaviors with the clinical periodontal parameters. The VIFs of the five salivary antioxidants variables in model I were 1.00~1.41. The VIFs of 13 variables (five salivary antioxidants variables, six conventional risk factor variables, and two oral behavior variables) in model II were 1.07–1.57. The statistical software G*Power was used to calculate the power analyses. A two-tailed multiple linear regression using random effects with thirteen predictors was employed, and the R^2^ for the full-model with the predictor variables was 0.13, 0.12, and 0.09, for the dependent variable of the PI, BOP, and PD mean, respectively. We assumed the probability of a type I error of 0.05. There were 225 patients’ data collected for this study and this yielded a power of around 0.95, 0.94, and 0.83 in testing hypotheses concerning all the conventional risk factors, oral health behavior and salivary oxidative stress biomarkers in the PI, BOP, and PD mean, respectively. Statistical significance was set to *p* < 0.05.

## 3. Results

Table 1 shows the demographic characteristics, oral health behaviors, and the distributions of the periodontal clinical parameters. The PI of the subjects who brushed their teeth more than twice daily was significantly lower than those subjects who brushed them once daily (53.46% vs. 69.29%, *p* < 0.01). Subjects with a lower educational level had a borderline significantly higher BOP than subjects with a higher education level (*p* = 0.05). There were no associations of demographic characteristics or oral health behaviors (tooth cleaning frequency or auxiliary tooth cleaning tools) with the BOP or PD mean. There were no statistically significant differences in the periodontal clinical parameters between subjects with irregular dental visits and subjects with regular dental visits.

Endogenous antioxidants were influenced by demographic characteristics and oral health behaviors. As shown in Table 2, the Cu/Zn SOD level of subjects with irregular dental visits was significantly lower than that of subjects with regular dental visits. The MnSOD level in betel nut chewers was lower than that in non-chewers. There were no associations of demographic characteristics or oral health behaviors (tooth cleaning frequency or auxiliary tooth cleaning tools) with salivary Prx2. Smokers and subjects whose last previous dental visit was <1 year had significantly lower Trx1 levels than non-smokers and subjects whose last previous dental visit was >1 year. Subjects who were male, brushed their teeth more than twice daily, and had regular dental visits had significantly lower CAT levels than subjects who were female, brushed once daily, and had irregular dental visits. In our study, the PI, BOP, and mean PD of the subjects with irregular dental visits were 60.40% ± 1.54%, 45.27% ± 1.77%, and 3.54 ± 0.04 mm, respectively, and were slightly higher than subjects who had regular dental visits (55.82% ± 2.02%, 40.05% ± 2.22%, and 3.44 ± 0.07 mm, respectively).

Table 3 shows the results of the univariate regression analysis of the conventional factors, oral health behaviors, salivary oxidative stress biomarkers, and clinical periodontal parameters. There were no associations of the conventional factors of gender, alcohol consumption, or betel nut chewing with clinical periodontal parameters. Borderline significant associations were shown between educational level and smoking status with BOP or the PD mean, as subjects with an educational level of university or above had a borderline significantly lower BOP (5.61%) and lower PD mean (0.13 mm) than subjects with an educational level of high school (*p* = 0.05 and *p* = 0.06, respectively). Smokers had a borderline significantly lower BOP (5.45%) than that of non-smokers (*p* = 0.07). The tooth cleaning frequency was associated with the PI, as subjects who brushed their teeth twice or more than twice daily had a significantly lower PI (9.35% and 15.83%) than that of subjects who brushed their teeth once daily (*p* = 0.02 and *p* < 0.001, respectively). Subjects who brushed their teeth more than twice daily had a significantly lower PI (6.47%) than that of subjects who brushed their teeth twice daily (*p* = 0.02 and *p* < 0.001, respectively). Auxiliary tooth cleaning tools were associated with the PI, as subjects who used floss or an interdental brush had a significantly lower PI (6.42%) than that of subjects who did not use floss or an interdental brush (*p* = 0.02). Subjects whose last previous dental visit was <1 year had a borderline significantly lower PI (−4.58%) and BOP (5.23%) than subjects whose last previous dental visit was >1 year (*p* = 0.08 and *p* = 0.08, respectively).

**Figure 2:**
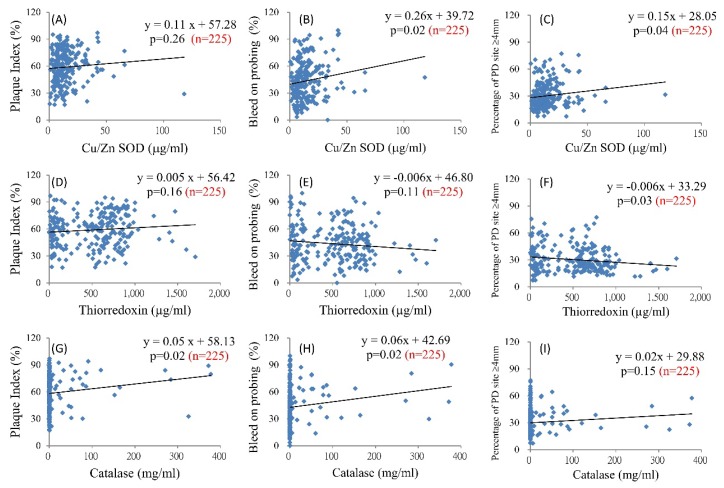
Association between the salivary antioxidants and clinical periodontal parameters. Salivary Cu/Zn superoxide dismutase (SOD) and dental clinical parameters (**A**–**C**); salivary thioredoxin 1 and dental clinical parameters (**D**–**F**); and salivary catalase and dental clinical parameters (**G**–**I**).

Salivary endogenous antioxidants may be associated with clinical periodontal parameters. Figure 2 shows the scatterplots of Cu/Zn SOD, Trx1, and CAT with PI, BOP, and the percentage of sites with a PD of ≥4 mm. Cu/Zn SOD significantly increased with increments in the BOP and percentage of sites with a PD of ≥4 mm (Figure 2B,C). With an increase of 1 μg/mL Cu/Zn SOD in the saliva, BOP significantly increased by 0.26% (*p* = 0.02, Figure 2B). Trx1 significantly increased when the percentage of sites with a PD of ≥4 mm decreased; with an increase in 1 μg/mL Trx1, a significant decrease of 0.006% was observed at sites with a PD of ≥4 mm (*p* = 0.03, Figure 2F). CAT significantly increased with an increment in the PI or BOP (Figure 2H,I). Associations of salivary MnSOD and Prx2 with the periodontal clinical parameters were non-significant.

Salivary endogenous antioxidants were shown to be statistically significantly associated with the pattern of dental visits (Table 2). To explore the associations of the pattern of dental visits with salivary endogenous antioxidants and periodontal clinical parameters, Table 4 and Table 5 show multiple regression models of salivary endogenous antioxidants and periodontal parameters in subjects whose last previous dental visit was >1 year and <1 year, respectively. All the dependent variables in models I and II were the clinical periodontal parameters. The five endogenous antioxidants exhibited sequential responses when exposed to oxidative stress. When one of the endogenous antioxidants was set as the independent variable, the other four endogenous antioxidants were adjusted in models I, III, and V. Table 4 shows the multiple regression analysis of salivary oxidative stress biomarkers and clinical periodontal parameters in subjects whose last previous dental visit was >1 year. After adjusting for the four other endogenous antioxidants, the PI, BOP, and PD mean of subjects whose last previous dental visit was >1 year significantly increased with a decrement in Cu/Zn SOD. With an increase of 1 µg/mL in Cu/Zn SOD, the PI significantly (*p* < 0.01) increased by 0.47% (model I), BOP significantly (*p* < 0.01) increased by 0.64% (model III), and the PD mean significantly (*p* = 0.04) increased by 8.37 × 10^−03^ mm (model V). The same phenomenon was reflected in models II, IV, and VI, which were adjusted for the four other endogenous antioxidants and conventional risk factors of periodontitis, such as gender, age, education, smoking status, alcohol consumption, betel nut chewing, tooth cleaning frequency, and auxiliary tooth cleaning tool types. In subjects whose last previous dental visit was >1 year, after adjusting for Cu/Zn SOD, MnSOD, Prx2, and CAT, the BOP significantly decreased by 0.02% when salivary Trx1 increased by 1 µg/mL (model III). The same phenomenon was shown in model IV (β = −0.02, *p* < 0.0001). In subjects whose last previous dental visit was >1 year, the PD mean significantly decreased with an increase in Trx1 (in both models V and VI).

Table 5 shows the multiple regression analysis of salivary oxidative stress biomarkers and clinical periodontal parameters in subjects whose last previous dental visit was <1 year. In subjects whose last previous dental visit was <1 year, after adjusting for the four other endogenous antioxidants, when salivary catalase increased by 1 mg/mL, the BOP significantly increased by 0.04% (*p* = 0.04, model III). The phenomenon changed to borderline non-significant when the conventional risk factors of periodontitis were adjusted for in model IV (β = 0.04, standard error (SE) = 0.02, *p* = 0.07). The main effect of the BOP percentage was on the conventional risk factors of periodontitis, not on the CAT. After adjusting for the four other endogenous antioxidants, the PD mean of subjects whose last previous dental visit was <1 year significantly decreased with an increment in MnSOD. With an increase of 1 µg/mL MnSOD, a significant (*p* = 0.02) decrease in the PD mean of 6.61 × 10^−3^ mm was observed (model V). The same phenomenon was shown in model VI, in which the four other endogenous antioxidants and conventional risk factors of periodontitis, and oral health behaviors were controlled for (β = −7.55 × 10^−3^, *p =* 0.01, model VI). In subjects whose last previous dental visit was <1 year, a higher salivary MnSOD was a protective factor against periodontitis. The PD mean significantly increased by 1.39 (*p* = 0.02) and 1.63 (*p* = 0.01) mm when the salivary CAT increased by 1 mg/mL (in models V and VI, respectively).

## 4. Discussion

This cross sectional study designed a multiple regression method looking at the effect of conventional risk factors (gender, education, smoking status, alcohol consumption, betel consumption); oral health behavior (tooth cleaning frequency, auxiliary tooth cleaning tools types, and dental visit patterns); and salivary oxidative stress biomarkers (Cu-Zn SOD, MnSOD, PRX2, TRX1 and Catalase) on the clinical periodontal parameters (PI, BOP, and PD mean) in periodontitis patients. In this study, associations of endogenous antioxidants with clinical periodontal parameters were investigated. After adjusting for other physical endogenous antioxidants and conventional risk factors, the strength of associations between endogenous antioxidants and clinical periodontal parameters in subjects whose last previous dental visit was <1 year was lower than that in subjects whose last previous dental visit was >1 year. Salivary antioxidants may serve as biomarkers to represent the disease status and ensure treatment effectiveness against periodontitis [26]. Excess hydrogen peroxide induces the production of Trx1 to resist oxidative stress [31]. Trx induces MnSOD when oxidative stress is produced. After SOD catalyzes superoxide anions to hydrogen peroxide, the hydrogen peroxide is subsequently catalyzed to oxygen and water by CAT or Trx [32]. Prx2 possesses a high-affinity binding site for hydrogen peroxide when a cell lacks CAT. CAT catalyzes hydrogen peroxide into oxygen and water [15,16]. The inflammatory status of periodontal tissues is caused by an oxidation-reduction imbalance and it is mediated by the endogenous antioxidant system [33,34]. The strength of the associations between endogenous antioxidants and the periodontal disease status can be disrupted by the pattern of dental visits.

The percentage of plaque present is a sensitive index that varies with food intake and oral hygiene practices. Periodontal disease can effectively be prevented by maintaining personal oral hygiene and scaling via regular dental visits [35]. The accumulation of dental plaque and calculus plays a critical role in periodontal inflammation [36]. Inflammation causes oxidative stress, and oxidative stress is eliminated by endogenous antioxidants. SOD is a coenzyme for converting superoxide into hydrogen peroxide and it exists in different organelles of mammalian cells. Of the three SOD enzymes, Cu/Zn SOD is in the cytosol, MnSOD is in mitochondria, and extracellular superoxide dismutase (ecSOD) is located in the extracellular matrix of tissues [37]. Previous studies showed that the gingival index was significantly associated with salivary SOD, and salivary SOD also significantly decreased in patients after periodontal disease treatment [26]. Our previous study also showed that SOD significantly increased with an increment in the BOP [38]. Endogenous antioxidants are associated with periodontitis as shown in this study.

Teeth brushing, dental flossing, and regular dental examinations are important behavioral indicators of good oral hygiene. Teeth brushing and flossing mainly clean plaque and reduce gum inflammation [39]. A meta-analysis study showed that subjects whose last previous dental visit was <1 year had a lower risk of periodontal disease than subjects whose last previous dental visit was >1 year (odds ratio: 0.27~0.68) [11]. A previous study showed no statistically significant differences in gingivitis between 6- and 12-month scale and polish intervals (with oral hygiene instruction) at time points of 24, 36, and 48 months [29]. There were no statistically significant differences in plaque at 3, 7, or 11 months, nor for measurements of loss of attachment after 11 months [28]. Suomi et al. reported no statistically significant differences in PDs between 4-month, 6-month, and 12-month scale and polish intervals (without oral hygiene instruction at 36 months [30]. Most of the significant differences were shown after the 1-year benchmark, because of the slow progression of periodontitis which affected all the indicators. Our previous study showed that total antioxidants in the saliva of subjects whose last previous dental visit was <1 year were higher than those in subjects whose last previous dental visit was >1 year [40]. Undergoing routine dental examinations is important for clinical periodontitis parameters in periodontitis patients. In this study, associations between endogenous antioxidants and clinical periodontal parameters in subjects whose last previous dental visit was >1 year were greater than those in subjects whose last dental visit was <1 year previous.

The physical response curve of endogenous antioxidants is bell-shaped [33]. In the early stage of the inflammatory response, endogenous antioxidants were positively correlated with the inflammatory status. BOP, known as gingival bleeding, reflects the inflammatory response when the gingiva is exposed to bacterial pathogens. BOP can also reflect the severity of inflammation and is an important index used to evaluate infection control during periodontitis treatment. In this study, Cu/Zn SOD significantly increased with the BOP and the percentage of sites with a PD of >4 mm. After adjusting for endogenous antioxidants and conventional risk factors, Cu/Zn SOD significantly increased with all clinical periodontal parameters in subjects whose last previous dental visit was >1 year. These results showed that an inflammatory effect was still present in the cytosol of subjects whose last previous dental visit was >1 year. In this study, lower Trx1 and CAT levels were seen in subjects whose last previous dental visit was <1 year. After adjusting for endogenous antioxidants and conventional risk factors, MnSOD significantly decreased with the percentage of sites with a PD of >4 mm in subjects whose last previous dental visit was <1 year. These results showed that mitochondrial antioxidants were related to clinical periodontal parameters in subjects whose last previous dental visit was <1 year.

In this study, Prx2 was not associated with clinical periodontal parameters, but Trx1 decreased with the percentage of sites with a PD of >4 mm in subjects whose last previous dental visit was >1 year. These results showed that Trx1 plays an antioxidant role in subjects whose last previous dental visit was >1 year. Trx1 synergizes with Prx to eliminate hydrogen peroxide [41]. With the last visit being <1 year previous, intracellular Trx1 expression is induced, and Trx1 reduces the translocation of nuclear factor (NF)-κB to nuclei via binding to NF-κB. Once translocation of NF-κB to nuclei is reduced, cytokine expressions are reduced [42]. Trx1 plays an important role in the endogenous antioxidant system in subjects whose last dental visit was >1 year.

The last dental visit being <1 year results in the modulation of several important physiological functions and periodontal disease activities. Excessive production of ROS leads to progressive oxidative damage via the response to periodontal injury and inflammation [43,44]. A previous study showed that cellular function was regulated by the redox status, and endogenous antioxidants could possibly be developed as drugs for treating inflammation-related diseases [33]. A recent study showed that salivary SOD significantly increased at 3 and 6 months in periodontitis subjects who received omega-3 for 6 months [45].

The current study had several limitations. First, a limitation of this study and of almost all studies of dental visit patterns is that the subject’s specific recall date of dental visits could not be tracked down through the medical system. Self-reporting bias could have occurred when dental visit frequency data were collected from the questionnaires in this study. In order to minimize or eliminate such bias, experienced and well-trained interviewers were used, and structured questionnaires were designed. Second, the associations between salivary antioxidants and clinical periodontitis parameters may have been affected by outliers. Stronger associations were found when the outliers were removed from the dataset in this study. Another possible limitation was that this study had limited statistical power for salivary MnSOD and Trx1, and the precision of our estimates was threatened by the small sample size. Therefore, our results for these two salivary markers and clinical periodontal parameters being related to dental visit patterns should be interpreted with caution. Despite the significant associations between the length of time since last the dental visit and salivary antioxidants, the major limitation of this study was that dental visits were influenced by other factors such as a patient’s socioeconomic background and oral health knowledge. Socioeconomic background and oral health knowledge also influenced salivary antioxidants via enhancing the knowledge and behaviors of nutritional intake. Further study is also required to demonstrate the mechanisms among endogenous antioxidants, oral health knowledge, oral hygiene, and routine dental checkups to enhance the prevention of periodontal disease.

## 5. Conclusions

This study showed that a longer duration between dental visits (>1 year) increased the association between salivary antioxidants and clinical periodontal parameters. The conclusion of this study merely suggests the possible correlation between antioxidant levels and free radical generation during the development of periodontitis in subjects who have longer intervals between dental visits. The precise level of free radical generation under this paradigm should be determined in future studies. Therefore, the home self-screening salivary antioxidant strips may elevate insights into periodontitis in subjects with dental phobias and compel those subjects to get regular dental checkups.

## Figures and Tables

**Table 1:** Demographic characteristics, oral health behaviors, and distributions of periodontal clinical parameters.

Periodontal Clinical Parameters	*N*	PI (%)	BOP (%)	PD (mm)
Variables		Mean ± SE		
Gender	225	58.88 ± 1.24	43.58 ± 1.4	3.44 ± 0.03
Female	122	58.12 ± 1.57	43.19 ± 1.77	3.41 ± 0.04
Male	103	59.77 ± 1.97	44.05 ± 2.25	3.48 ± 0.05
*p* value ^a^		0.51	0.76	0.34
Educational level				
High school	90	59.17 ± 1.89	46.97 ± 2.16	3.52 ± 0.06
University or above	135	58.68 ± 1.64	41.36 ± 1.83	3.39 ± 0.04
*p* value ^a^		0.84	0.05	0.06
Smoking status				
Non-smoker	159	59.74 ± 1.46	45.15 ± 1.66	3.43 ± 0.04
Smoker	66	56.8 ± 2.33	39.7 ± 2.6	3.48 ± 0.07
*p* value ^a^		0.28	0.07	0.55
Alcohol consumption				
Never or occasional	200	58.55 ± 1.31	43.28 ± 1.49	3.43 ± 0.03
Regular	25	61.46 ± 3.93	46.03 ± 4.22	3.55 ± 0.12
*p* value ^a^		0.46	0.54	0.25
Betel nut chewing				
Non-chewer	218	58.62 ± 1.27	43.54 ± 1.42	3.45 ± 0.03
Chewer	7	66.93 ± 4.87	44.9 ± 10.33	3.3 ± 0.15
*p* value ^a^		0.24	0.86	0.45
Tooth cleaning frequency				
Once per day	23	69.29 ± 4.21	46.04 ± 5.65	3.54 ± 0.11
Twice per day	132	59.93 ± 1.54	42.61 ± 1.7	3.42 ± 0.04
More than twice per day	70	53.46 ± 2.16	44.6 ± 2.61	3.46 ± 0.07
*p* value ^b^		<0.01	0.69	0.59
Auxiliary tooth cleaning tools				
None	62	63.51 ± 2.36	47.5 ± 2.98	3.52 ± 0.07
Floss or interdental brush	118	57.08 ± 1.72	41.16 ± 1.75	3.41 ± 0.04
Floss and interdental brush	45	57.19 ± 2.6	44.52 ± 3.31	3.44 ± 0.08
*p* value ^b^		0.07	0.15	0.34
Length of time since last dental visit				
>1 year	150	60.4 ± 1.55	45.28 ± 1.77	3.45 ± 0.04
<1 year	75	55.82 ± 2.02	40.05 ± 2.23	3.44 ± 0.07
*p* value ^a^		0.08	0.08	0.97

PI, plaque index; BOP, bleeding on probing; PD, probing depth; SE, standard error. ^a^ *p* value for Student’s *t*-test; ^b^ *p* value for an ANOVA.

**Table 2:** Demographic characteristics, oral health behaviors, and distributions of salivary antioxidants.

Salivary Antioxidant	*N*	Cu/Zn SOD (µg/mL)	MnSOD (µg/mL)	Prx2 (µg/mL)	Trx1 (µg/mL)	Catalase (mg/mL)
Variable		Mean ± SE				
All subjects	225	14.87 ± 0.87	17.8 ± 2.38	4.81 ± 1.36	504.88 ± 23.83	14.25 ± 3.52
Gender						
Female	122	13.79 ± 0.85	18.18 ± 3.2	2.64 ± 0.53	509.23 ± 32.16	22.08 ± 6.25
Male	103	16.14 ± 1.61	17.34 ± 3.58	7.38 ± 2.89	499.73 ± 35.65	4.98 ± 1.72
*p* value ^a^		0.19	0.86	0.11	0.84	<0.01
Educational level						
High school	90	13.33 ± 1.12	11.62 ± 2.7	3.13 ± 0.77	448.72 ± 33.86	23.09 ± 7.41
University or above	135	15.89 ± 1.25	21.91 ± 3.51	5.93 ± 2.21	542.32 ± 32.38	8.35 ± 3.09
*p* value ^a^		0.12	0.02	0.23	0.05	0.07
Smoking status						
Non-smoker	159	15.62 ± 1.07	18.19 ± 2.87	5.84 ± 1.9	540.02 ± 27.78	16.93 ± 4.83
Smoker	66	13.05 ± 1.46	16.86 ± 4.3	2.34 ± 0.6	420.23 ± 44.67	7.78 ± 2.79
*p* value ^a^		0.18	0.80	0.08	0.02	0.10
Alcohol consumption						
Never or occasional	200	14.91 ± 0.92	18.17 ± 2.62	3.56 ± 0.98	517.67 ± 25.2	14.88 ± 3.93
Regular	25	14.56 ± 2.77	14.84 ± 4.82	14.82 ± 9.3	402.55 ± 71.24	9.23 ± 4.14
*p* value ^a^		0.90	0.54	0.23	0.12	0.32
Betel nut chewing						
Non-chewer	218	15 ± 0.88	18.16 ± 2.46	4.89 ± 1.4	509.24 ± 24.06	14.47 ± 3.63
Chewer	7	10.63 ± 5.51	6.48 ± 2.18	2.48 ± 1.39	369.03 ± 161.87	7.48 ± 7.22
*p* value ^a^		0.38	<0.01	0.23	0.30	0.40
Tooth cleaning frequency						
Once per day	23	15.61 ± 2.68	15.97 ± 6.55	12.38 ± 9.86	522.5 ± 81.15	9.05 ± 4.84
Twice per day	132	13.77 ± 0.86	16.91 ± 3.12	3.95 ± 1.44	502.27 ± 30.57	11.77 ± 4.09
More than twice per day	70	16.7 ± 2.11	20.08 ± 4.44	3.94 ± 1.16	504.02 ± 43.45	20.62 ± 8.13
*p* value ^b^		0.30	0.80	0.17	0.96	0.46
Auxiliary tooth cleaning tools						
None	62	16.74 ± 1.48	26.83 ± 5.84	5.76 ± 3.68	445.71 ± 48.68	29.01 ± 10.18
Floss or interdental brush	118	14.77 ± 1.38	14.38 ± 2.85	3.11 ± 0.72	553.09 ± 31.48	5.98 ± 1.98
Floss and interdental brush	45	12.56 ± 1.26	14.31 ± 4.41	7.97 ± 4.15	460 ± 52.22	15.6 ± 8.89
*p* value ^b^		0.26	0.06	0.36	0.10	0.02
Length of time since last dental visit						
>1 year	150	12.98 ± 0.74	15.57 ± 2.81	3.52 ± 1.58	575.11 ± 28.18	21.24 ± 5.19
<1 year	75	18.64 ± 2.11	22.26 ± 4.4	7.4 ± 2.58	364.44 ± 39.48	0.26 ± 0.02
*p* value ^a^		0.01	0.18	0.17	<0.0001	<0.0001

^a^*p* value for Student’s *t*-test; ^b^ *p* value for an ANOVA; SE, standard error; SOD superoxide dismutase; Prx peroxiredoxin, Trx, thioredoxin.

**Table 3:** Univariate regression analysis of conventional factors, oral health behaviors, salivary oxidative stress biomarkers, and clinical periodontal parameters.

	Dependent Variable	PI (%)			BOP (%)			PD Mean (mm)		
Independent Variable		β	SE	*p* Value	β	SE	*p* Value	β	SE	*p* Value
Gender (male vs. female), *N* = 225		1.65	2.48	0.50	0.86	2.82	0.76	0.06	0.06	0.33
Education, *N* = 225 (university or above vs. high school)		−0.48	2.53	0.84	−5.61	2.85	0.05	−0.13	0.07	0.06
Smoking status, *N* = 225 (smokers vs. non-smokers)		−2.93	2.71	0.28	−5.45	3.08	0.07	0.04	0.07	0.55
Alcohol consumption, *N* = 225 (regular vs. never or occasional)		2.9	3.94	0.46	2.74	4.53	0.54	0.12	0.11	0.25
Betel nut chewing, *N* = 225 (chewers vs. non-chewers)		8.31	7.12	0.24	1.36	8.05	0.85	−0.14	0.2	0.45
Tooth cleaning frequency										
Twice per day vs. once per day, *N* = 155		−9.35	4.04	0.02	−3.42	4.67	0.46	−0.11	0.11	0.29
More than twice per day vs. once per day, *N* = 93		−15.83	4.46	<0.001	−1.43	5.56	0.79	−0.08	0.13	0.54
More than twice per day vs. twice per day, *N* = 202		−6.47	2.63	0.01	1.99	3.00	0.50	0.03	0.08	0.65
Floss or interdental brush vs. none, *N* = 180		−6.42	2.93	0.02	−6.34	3.24	0.05	−0.12	0.0.8	0.13
Floss and interdental brush vs. none, *N* = 107		−6.32	3.54	0.07	−2.98	4.48	0.50	−0.07	0.11	0.45
Floss and interdental brush vs. floss or interdental brush, *N* = 163		0.11	3.22	0.97	3.36	3.48	0.33	0.04	0.09	0.66
Length of time since last dental visit (<1 year vs. >1 year), *N* = 225		−4.58	2.61	0.08	−5.23	2.98	0.08	−2.52 × 10^−3^	0.07	0.97

β, estimated coefficient; SE, standard error; PI, plaque index; BOP, bleeding on probing; PD, pocket depth.

**Table 4:** Multiple regression analysis of salivary oxidative stress biomarkers and clinical periodontal parameters in subjects whose last previous dental visit was >1 year (*N* = 150).

	Dependent Variable: PI (%)	Model I ^§^			Model II ^¶^		
Independent Variable		β	SE	*p* Value	β	SE	*p* Value
Cu-Zn SOD (µg/mL)		0.47	0.18	<0.01	0.38	0.18	0.04
MnSOD (µg/mL)		−0.03	0.05	0.05	−0.02	0.05	0.64
Prx2 (µg/mL)		0.05	0.08	0.56	−0.01	0.08	0.92
Trx1 (µg/mL)		5.14 × 10^−3^	4.46 × 10^−3^	0.25	3.89 × 10^−3^	4.45 × 10^−3^	0.38
Catalase (mg/mL)		0.05	0.03	0.07	0.05	0.02	0.04
	**Dependent variable: BOP (%)**	**Model III ^§^**			**Model IV ^¶^**		
**Independent variable**		**β**	**SE**	***p* value**	**β**	**SE**	***p* value**
Cu-Zn SOD (µg/mL)		0.64	0.19	<0.001	0.56	0.19	<0.01
MnSOD (µg/mL)		0.03	0.05	0.51	0.05	0.05	0.35
Prx2 (µg/mL)		0.10	0.08	0.23	0.05	0.09	0.54
Trx1 (µg/mL)		−0.02	4.75 × 10^−3^	<0.001	−0.02	4.82 × 10^−3^	<.0001
Catalase (mg/mL)		0.04	0.02	0.09	0.04	0.03	0.15
	**Dependent variable: PD mean (mm)**	**Model V ^§^**			**Model VI ^¶^**		
**Independent variable**		**β**	**SE**	***p* value**	**β**	**SE**	***p* value**
Cu-Zn SOD (µg/mL)		8.37 × 10^−3^	4.07 × 10^−3^	0.04	7.42 × 10^−3^	4.25 × 10^−3^	0.08
MnSOD (µg/mL)		5.12 × 10^−4^	1.08 × 10^−3^	0.64	7.96 × 10^−4^	1.11 × 10^−3^	0.47
Prx2 (µg/mL)		3.08 × 10^−3^	1.82 × 10^−3^	0.09	1.47 × 10^−3^	1.93 × 10^−3^	0.45
Trx1 (µg/mL)		−4.91 × 10^−4^	1.03 × 10^−4^	<0.0001	−4.89 × 10^−4^	1.05 × 10^−4^	<0.0001
Catalase (mg/mL)		6.51 × 10^−4^	5.54 × 10^−4^	0.24	6.54 × 10^−4^	5.82 × 10^−4^	0.26

β, estimated coefficient; SE, standard error; PI, plaque index; BOP, bleeding on probing; PD pocket depth; SOD, superoxide dismutase; Prx, peroxiredoxin; Trx, thioredoxin. ^§^ Model included Cu-Zn SOD, MnSOD, Prx2, Trx1, and catalase. ^¶^ Model included Cu-Zn SOD, MnSOD, Prx2, Trx1, catalase, and conventional risk factors (gender, age, educational level, smoking status, alcohol consumption, betel nut consumption, tooth cleaning frequency, and auxiliary tooth cleaning tool types).

**Table 5:** Multiple regression analysis of salivary oxidative stress biomarkers and clinical periodontal parameters in subjects whose last previous dental visit was <1 year (*N* = 75).

	Dependent Variable: PI (%)	Model I ^§^			Model II ^¶^		
Independent Variable		β	SE	*p* Value	β	SE	*p* Value
Cu-Zn SOD (µg/mL)		−0.03	0.20	0.87	3.54 × 10^−3^	0.21	0.99
MnSOD (µg/mL)		−0.14	0.09	0.11	−0.14	0.09	0.12
Prx2 (µg/mL)		−0.14	0.10	0.16	−0.17	0.09	0.09
Trx1 (µg/mL)		1.14 × 10^−3^	0.01	0.91	−1.67 × 10^−3^	0.01	0.87
Catalase (mg/mL)		0.03	0.02	0.07	0.03	0.01	0.08
	**Dependent variable: BOP (%)**	**Model III ^§^**			**Model IV ^¶^**		
**Independent variable**		**β**	**SE**	***p* value**	**β**	**SE**	***p* value**
Cu-Zn SOD (µg/mL)		−0.05	0.22	0.82	−0.07	0.24	0.79
MnSOD (µg/mL)		−0.16	0.09	0.09	−0.14	0.11	0.19
Prx2 (µg/mL)		−0.10	0.10	0.31	−0.11	0.11	0.30
Trx1 (µg/mL)		0.01	0.01	0.30	0.01	0.01	0.41
Catalase (mg/mL)		0.04	0.02	0.04	0.04	0.02	0.07
	**Dependent variable: PD mean (mm)**	**Model V ^§^**			**Model VI ^¶^**		
**Independent variable**		**β**	**SE**	***p* value**	**β**	**SE**	***p* value**
Cu-Zn SOD (µg/mL)		2.08 × 10^−3^	6.65 × 10^−3^	0.76	3.71 × 10^−3^	6.79 × 10^−3^	0.59
MnSOD (µg/mL)		−6.61 × 10^−3^	2.80 × 10^−3^	0.02	−7.55 × 10^−3^	2.92 × 10^−3^	0.01
Prx2 (µg/mL)		−1.18 × 10^−3^	3.08 × 10^−3^	0.70	3.51 × 10^−4^	3.15 × 10^−3^	0.91
Trx1 (µg/mL)		1.36 × 10^−4^	3.26 × 10^−4^	0.68	4.93 × 10^−5^	3.32 × 10^−4^	0.88
Catalase (mg/mL)		1.39	0.60	0.02	1.63	0.61	0.01

β, estimated coefficient; SE, standard error; PI, plaque index; BOP, bleeding on probing; PD, pocket depth; SOD, superoxide dismutase; Prx, peroxiredoxin; Trx, thioredoxin. ^§^ Model included Cu-Zn SOD, MnSOD, Prx2, Trx1, and catalase. ^¶^ Model included Cu-Zn SOD, MnSOD, Prx2, Trx1, catalase, and conventional risk factors (gender, age, educational level, smoking status, alcohol consumption, betel nut consumption, tooth cleaning frequency, and auxiliary tooth cleaning tool types).
